# Vaginal delivery in a patient with severe aortic stenosis under epidural analgesia, a case report

**DOI:** 10.1186/s12947-020-00226-x

**Published:** 2020-11-02

**Authors:** Lorenza Driul, Francesco Meroi, Alessia Sala, Silvia Delrio, Daisy Pavoni, Federico Barbariol, Ambrogio Londero, Teresa Dogareschi, Alessandra Spasiano, Luigi Vetrugno, Tiziana Bove

**Affiliations:** 1 Department of Medicine, Gynecology and Obstetrics Clinic, University of Udine, Via Colugna n° 50, 33100 Udine, Italy; 2 Department of Maternal and Child Health, University-Hospital of Udine, P.le S. Maria della Misericordia n° 15, 33100 Udine, Italy; 3 Department of Medicine, Anesthesia and Intensive Care Clinic, University of Udine, Via Colugna n° 50, 33100 Udine, Italy; 4 Department of Anesthesia and Intensive Care, University-Hospital of Udine, P.le S. Maria della Misericordia n° 15, 33100 Udine, Italy; 5 Department of Cardiothoracic Sciences, University-Hospital of Udine, P.le S. Maria della Misericordia n° 15, 33100 Udine, Italy

**Keywords:** Severe aortic stenosis, Labor induction, Epidural anesthesia

## Abstract

**Background:**

A history of previous cardiac disease increases the maternal mortality risk by as much as 100%. There is no consensus on the absolute contraindications to vaginal delivery in valvular heart disease, but central regional anesthesia is traditionally considered contraindicated in patients with severe aortic stenosis.

**Case presentation:**

A 29-year-old primigravid woman with severe aortic stenosis was admitted to the obstetrics department for programmed labor induction. With epidural anesthesia and mini-invasive hemodynamic monitoring labor and operative vaginal delivery were well tolerated, and hemodynamic stability was always maintained.

**Conclusions:**

Epidural analgesia and oxytocin induction are possible for the labor management of parturients with severe aortic stenosis given that continuous non-invasive followed by invasive hemodynamic monitoring can be provided and given the absence of any obstetric or cardiologic contraindications and the strong will of the patient.

**Supplementary Information:**

**Supplementary information** accompanies this paper at 10.1186/s12947-020-00226-x.

## Background

The management of pregnant patients with a history of cardiac disease is challenging even for expert anesthesiologists. Although the prevalence of clinically relevant heart disease is relatively low, occurring in approximately 1% of pregnancies, major cardiac events occur in up to 13% of pregnant patients with history of previous cardiac disease [1]. Valvular heart disease accounts for approximately 40% of these cases. The presence of a bicuspid aortic valve with/without stenosis or insufficiency is the most prevalent defect, and represents the most common indication for cardiac surgery in women of a fertile age [2].

Briefly from a physiological stand point, hormonal shifts during pregnancy (specifically, the rise in progesterone and prostacyclin) bring about significant hemodynamic changes: systemic vascular resistance and blood pressure decrease, whereas heart rate and stroke volume increase, determining a rise in cardiac output [3].

The risk of cardiac complications is greatest during the peripartum period, when factors such as uterine contractions, pain, fatigue, bleeding, uterine involution and anesthesia/analgesia may cause significant shifts in hemodynamics, leading to acute heart failure [4]. There is no consensus on the absolute contraindications to vaginal delivery in valvular heart disease, but central regional anesthesia is traditionally considered contraindicated in patients with severe aortic stenosis [5].

Here we report on the management of a pregnant woman with severe aortic stenosis who underwent labor with epidural analgesia and close hemodynamic monitoring.

## Case presentation

**Fig. 1:**
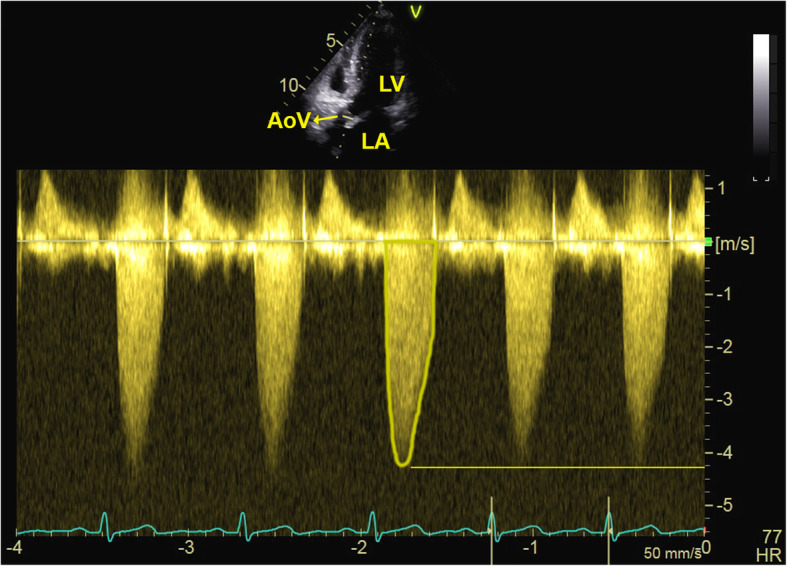
Biological prosthesis aortic valve pressure gradient


A 29-year-old primigravid woman with severe aortic stenosis was admitted to the obstetrics department for programmed labor induction. She weighed 54 Kilograms (Kg), was 160 cm (cm) high, and had no allergies. The patient had undergone valvuloplasty at the age of 2 years for the correction of congenital aortic stenosis, followed by total valve replacement (with a biological prosthesis) at the age of 21. Over the last 2 years (prior to and during her pregnancy), the patient had presented episodes of supraventricular tachycardia/atrial fibrillation, treated successfully with flecainide. At the last echocardiography performed at 36 weeks and 4 days gestation, she presented a left ventricle with normal dimensions, thickness and systolic function, a biological prosthesis on site with a thick and less mobile right coronary cusp, and normal motility of the left coronary and non-coronary cusps (Videos 1 and 2) with a maximum pressure gradient of 52 mmHg and an average of 31 mmHg (Fig. 1).

**Additional file 1.**


**Additional file 2.**

**Fig. 2:**
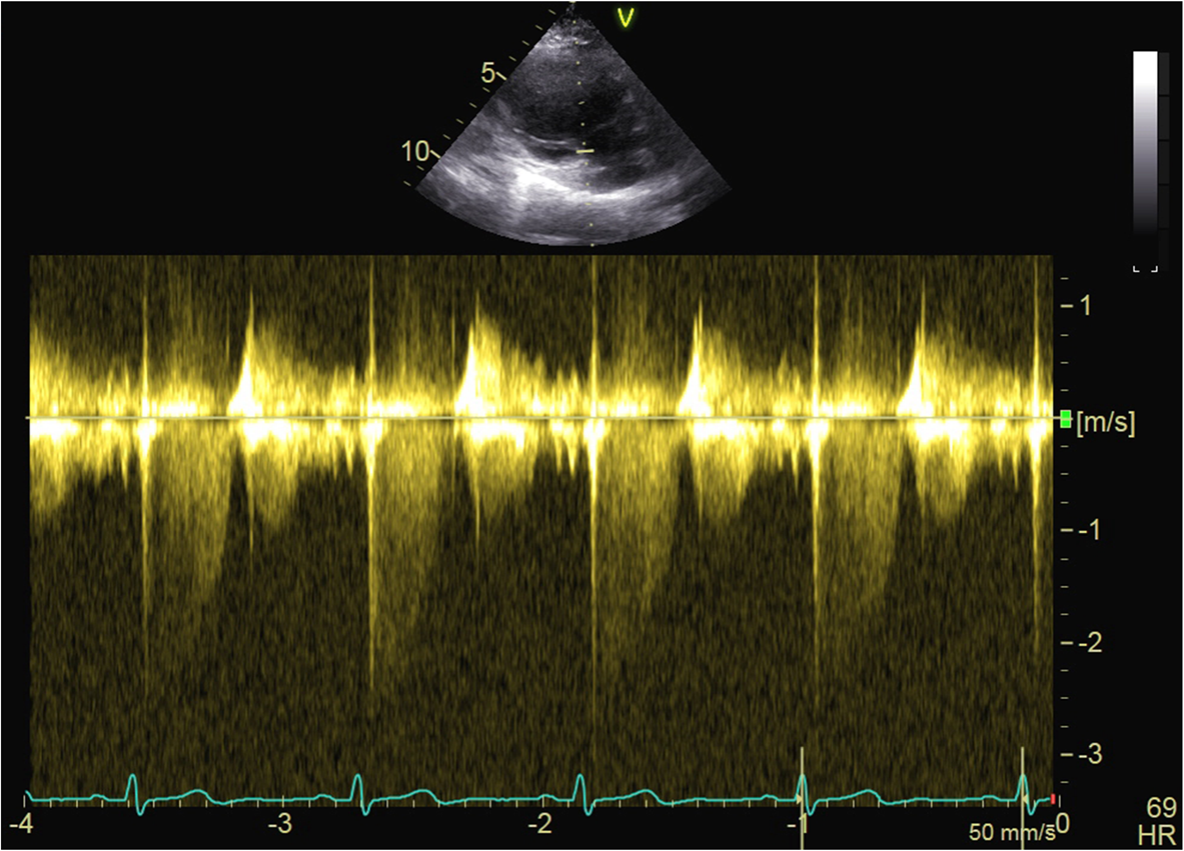
Pulmonary artery systolic pressure

**Fig. 3:**
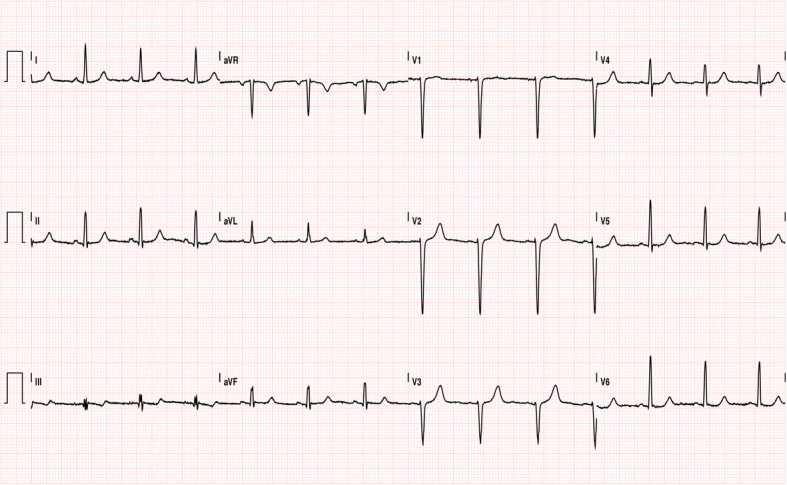
Electrocardiogram

The aortic valve area (AVA) was 0.9–1.0 cm^2^. She presented mild mitral insufficiency, an estimated pulmonary systolic pressure of 27 mmHg and a normal left atrium (Fig. 2). Electrocardiography revealed a normal sinus rhythm (Fig. 3). The patient was medicated with enoxaparin, 4000 international units (IU), twice daily.

The patient was followed by the ‘high-risk pregnancy service’ provided by our institution. Gestational age was calculated at the first trimester ultrasound scan. Her risk for chromosomal anomalies was low, and early and late fetal anomaly scans described normal fetal anatomy. An ultrasound scan performed at 35 weeks and 5 days gestation revealed normal fetal parameters and growth and an estimated fetal weight of 2888 g (65th percentile). A multidisciplinary team followed the patient, providing tailored therapy (anticoagulation), and following evaluation (a detailed obstetric visit and fetal weight estimation) the date of delivery was set to 38 weeks and 4 days of gestation. Considering the clinical history of the patient and her own preferences, and in accordance with the obstetricians, we opted for epidural labor analgesia. On the morning of the programmed labor induction, the patient’s vital parameters were monitored: electrocardiography (ECG), oxygen saturation (SpO_2_), non-invasive blood pressure (NIBP), and cardiotocography. A defibrillator was ready and nearby. We placed two large-bore intravenous catheters and started infusion with Ringer’s acetate solution. We then added a non-invasive hemodynamic monitoring system, using the ClearSight™ and EV1000 Platform (Edwards Lifesciences, Irvine, California, USA).

**Fig. 4:**
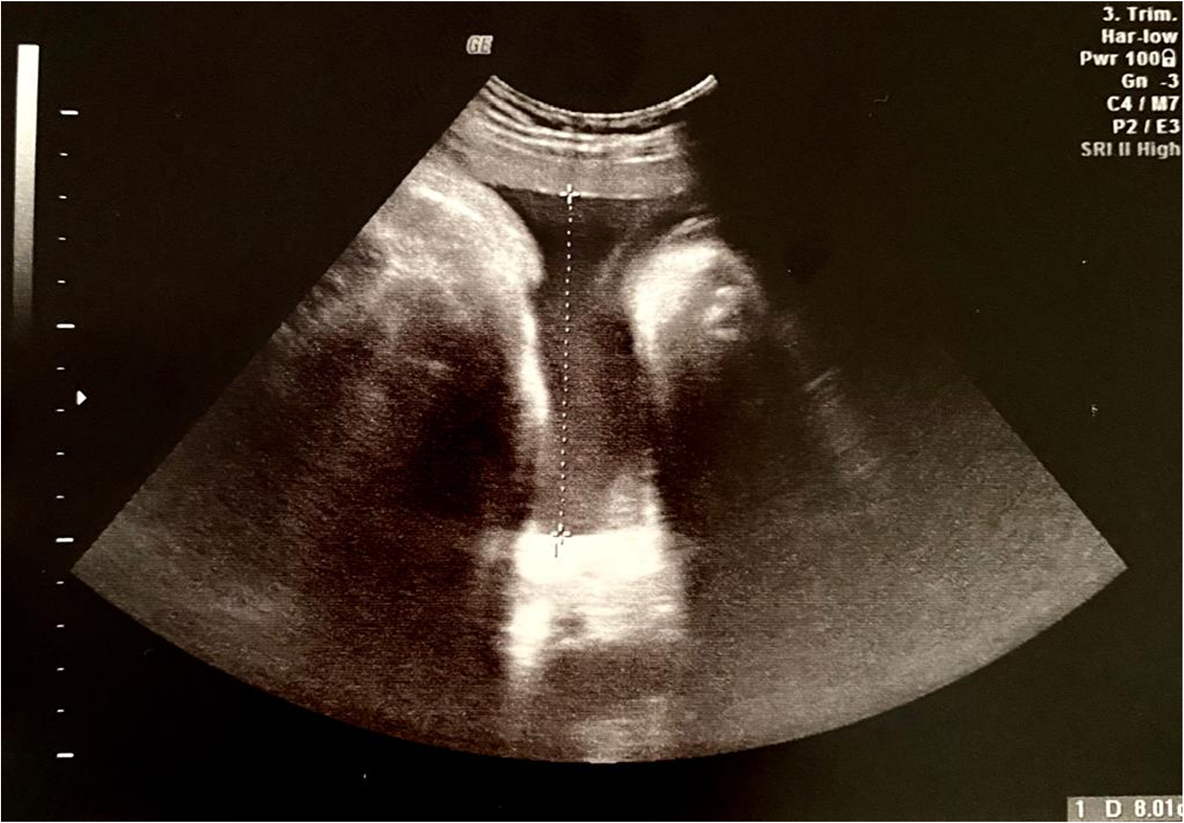
Regular amniotic fluid and a maximal vertical pocket of 80 mm

**Fig. 5:**
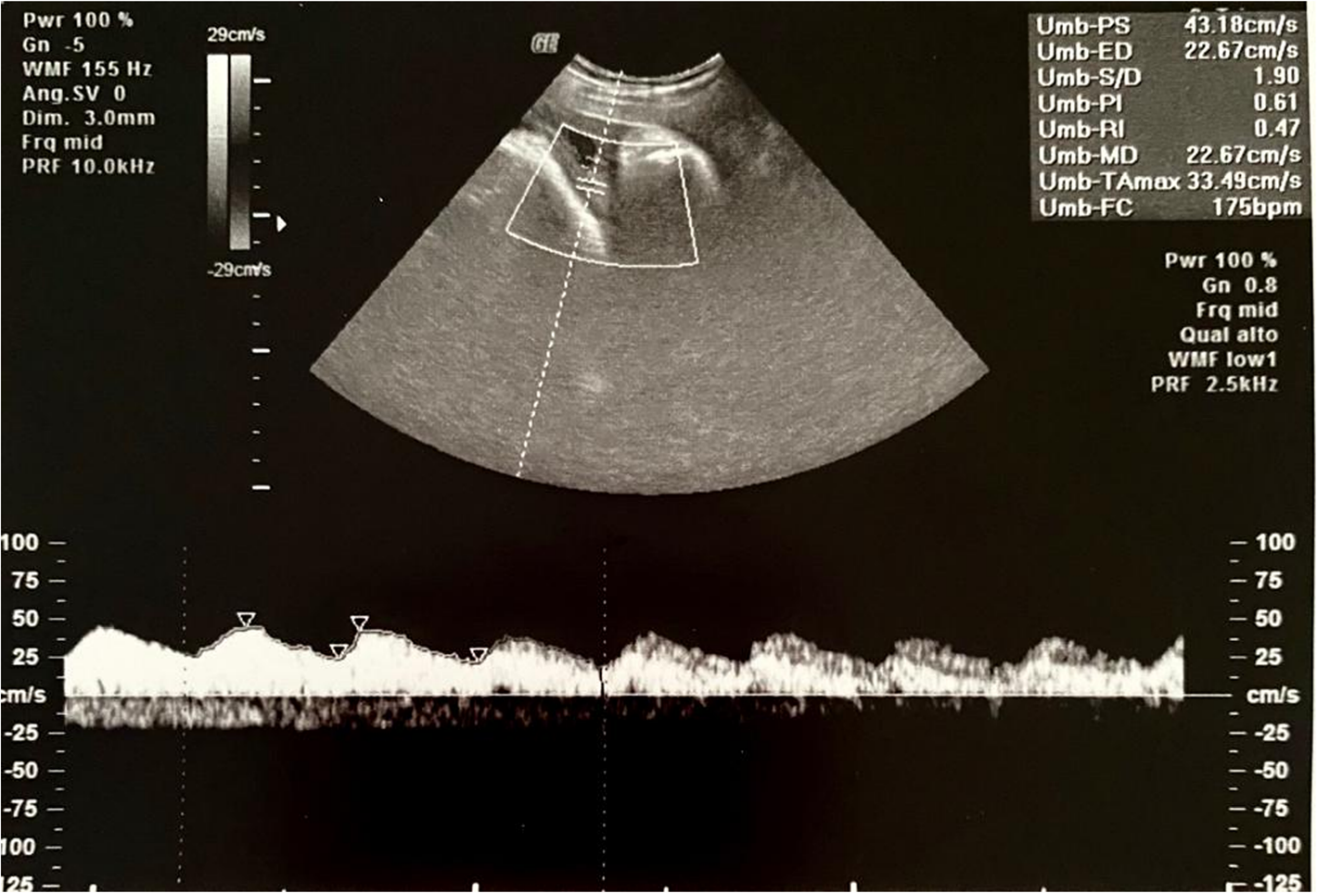
Umbilical artery pulsatility index

A trans-abdominal ultrasound scan with a convex probe 3.5 MHz (Voluson E, GE Healthcare, Chicago, Illinois, USA) showed a cephalic presentation, a maximal vertical pocket (DVP) of 80 mm, and an umbilical artery pulsatility index (UA PI) of 0.80 (Figs. 4 and 5). Vaginal examination revealed the cervix to be 2 cm dilated, 50% effaced, and to have a Bishop Score of 6. The patient’s baseline vital parameters were: heart rate (HR), 82 beats per minute (bpm); median blood pressure (mAP), 85 mmHg; SpO_2_, 100%; fetal heart rate (FHR), 159 bpm; cardiac index (CI), 4 L/min/m^2^; stroke volume index (SVI), 56 ml/m^2^.

Her laboratory exams reported: 11.1 g/dL hemoglobin; 220.000 platelets; international normalized ratio (INR), 0.91; activated partial thromboplastin time ratio (aPTTr), 0.89; fibrinogen, 551 mg/dL. Enoxaparin therapy was suspended 24 h before induction.

Using an 18-gauge (G) Tuohy needle, an epidural 20 G catheter (B. Braun, Melsungen, Germany) was placed into lumbar (L) vertebral space 2–3 and tested with ropivacaine 0.2%, 5 mL, fractionated into small boluses. All vital parameters were constantly monitored and induction started with a low dose oxytocin infusion (Table 1).

**Table 1:** Hemodynamic monitoring parameters recorded at: T0 = baseline, T1 = first epidural bolus, T2 = amniotomy, T3 = second epidural bolus, T4 = third epidural bolus, T5 = complete dilation, T6 = fourth epidural bolus, T7 = delivery, T8 = 30 min after delivery, T9 = 5 h after delivery, T10 = 10 h after delivery. HR = heart rate, MAP = median arterial pressure, CI = cardiac index, SVI = stroke volume index, FHR = fetal heart rate, CD = cervical dilation, CE = cervical effacement, OXY = oxytocin, FS = fetal station

	HR (bpm)	MAP (mmHg)	CI (L/min/m2)	SVI (mL/m2)	FHR (bpm)	CD (cm)	CE (%)	OXY (mUI/min)	FS (cm)
T0	82	85	4	56	159	2	50		
T1	77	90	4.2	55	141	4	80	2.5	
T2	69	107	4.2	60	140	5	80	7.5	
T3	79	94	4.9	63	146	8	100	10	−2
T4	71	83	5.4	76	145	9	100	12.5	−1
T5	92	102	4.9	53	145	10		15	
T6	74	99	4	55	147	10		17.5	
T7	114	111	4.4	38					+ 1
T8	78	110	3.6	46					
T9	71	102	3.5	50					
T10	74	105	3.4	45					

Five hours later, good progression of the first stage of labor was evident, with 5 cm cervical dilation and good effacement. An amniotomy was performed at this point. The patient gave a pain rating of 5 out of 10 (using the numeric pain rating scale, NPRS) and her hemodynamic parameters were stable. We started with an epidural infusion of ropivacaine 0.1%, 5 mL, and sufentanil, 3.75 mcg. After 30 min, the patient had a dermatomal level around thoracic (T) spinal nerve T10 (Table 2).

**Table 2:** Labor analgesia protocol

Test dose after epidural catheter positioning		Lidocaine 1%, 4 mL
First bolus	Latent phase of labor	Fentanyl 30–50 mcg (or sufentanil 5–7.5 mcg). Total volume 10 mL
	Early labor stage	Levobupivacaine 0.0625–0.1% (or ropivacaine 0.1–0.15%) + fentanyl 30 mcg (or sufentanil 5–7.5 mcg). Total volume 15–20 mL in 10 min
	Active labor or labor of a multipara	Levobupivacaine 0.1–0.125% (or ropivacaine 0.15–0.2%). Total volume 15–20 mL in 10 min
Following doses		Levobupivacaine 0.1% (or ropivacaine 0.1–0.15%) + fentanyl 1 mcg/mL (or sufentanil 0.25 mcg/mL). Total volume 15–20 mL
During delivery, before episiotomy, before operative vaginal delivery		Lidocaine 1%, 10 mL

During the following hours, we maintained an acceptable level of analgesia, a constant dermatomal level, and stable hemodynamic parameters with small boluses of ropivacaine 0.1% and sufentanil, ranging from 5 to 10 mL. During this period, the dose of oxytocin was increased by the obstetrician, and cervical dilation proceeded until complete dilation. At this point, in order to guarantee accurate hemodynamic monitoring, an artery line was put in place and connected to the FloTrac™ system (Edwards Lifesciences, Irvine, California, USA).

Three hours later, following episiotomy, the fetus was extracted using a vacuum (Kiwi Omni-C Cup, Ri.mos., Italy) to avoid excessive exertion by the mother and in response to initial alterations in the fetal cardiotocograph. Pain was controlled with alkalinized lidocaine 2%, 4 mL, given before extraction, and all vital and hemodynamic parameters remained stable (Table 1). A male baby weighing 3270 g was delivered with Apgar scores of 8 and 8 at minute 1 and 5, respectively. Blood loss did not exceed 100 mL. All the patient’s vital parameters were monitored for the next 10 h; we then removed the epidural catheter and all other monitoring devices and the patient was dismissed to the obstetrics ward with stable parameters. Pain was treated with 1000 mg paracetamol and 600 mg ibuprofen every 8 h, with an NRS < 3.

The post-partum period was characterized by episodes of fever and a suspected phlebitis of the hand. Considering the high cardiovascular risk of the patient, the infectologist suggested antibiotic therapy with daptomycin and piperacillin/tazobactam, then de-escalated to amoxicillin-clavulanic acid for a total of 20 days. One week after labor, echocardiography was repeated and shown to be equivalent to the one performed pre-partum. The patient was discharged from the hospital 11 days post-partum.

## Discussion

A history of previous cardiac disease increases the maternal mortality risk by as much as 100% [6]. The most common etiologies include congenital heart disease (~ 60%), valvular heart disease (~ 30%), and cardiomyopathies (~ 6%), whereas ischemic heart disease, aortopathies, and pulmonary hypertension account for a minority of cases (~ 4–5%) [7]. Peripartum cardiomyopathy is an idiopathic and rare heart disease that manifests itself in the late phases of pregnancy or early post-partum [8], with an incidence of one in 968 live births in American women [9]. It can clinically present with congestion symptoms, such as dyspnea on exertion, paroxysmal nocturnal dyspnea, orthopnea, and edema of the lower extremities [10]. Cardiogenic shock occurs in a small percentage of cases and less frequently unstable arrhythmias and arterial thromboembolism [8, 11, 12]. Left- and right-sided congestion are the typical clinical signs [8] and echocardiography is the best diagnostic tool [13].

All women of a fertile age with a history of heart disease should undergo pre-conception risk-assessment and counseling in centers with high expertise for cardiac diseases in pregnancy. Risk assessment should include history and physical examination, 12-lead ECG, and echocardiography. A cardiac CT scan or MRI and exercise testing may be required in specific cases [14].

The most widely used risk assessment score is the modified version of the WHO classification, which takes into account the specific cardiac lesion and divides patients into four classes of risk. Class I patients have no/mild risk, class II and III patients have intermediate/high risk, and class IV patients are at very high risk (pregnancy contraindicated) [4].

**Fig. 6:**
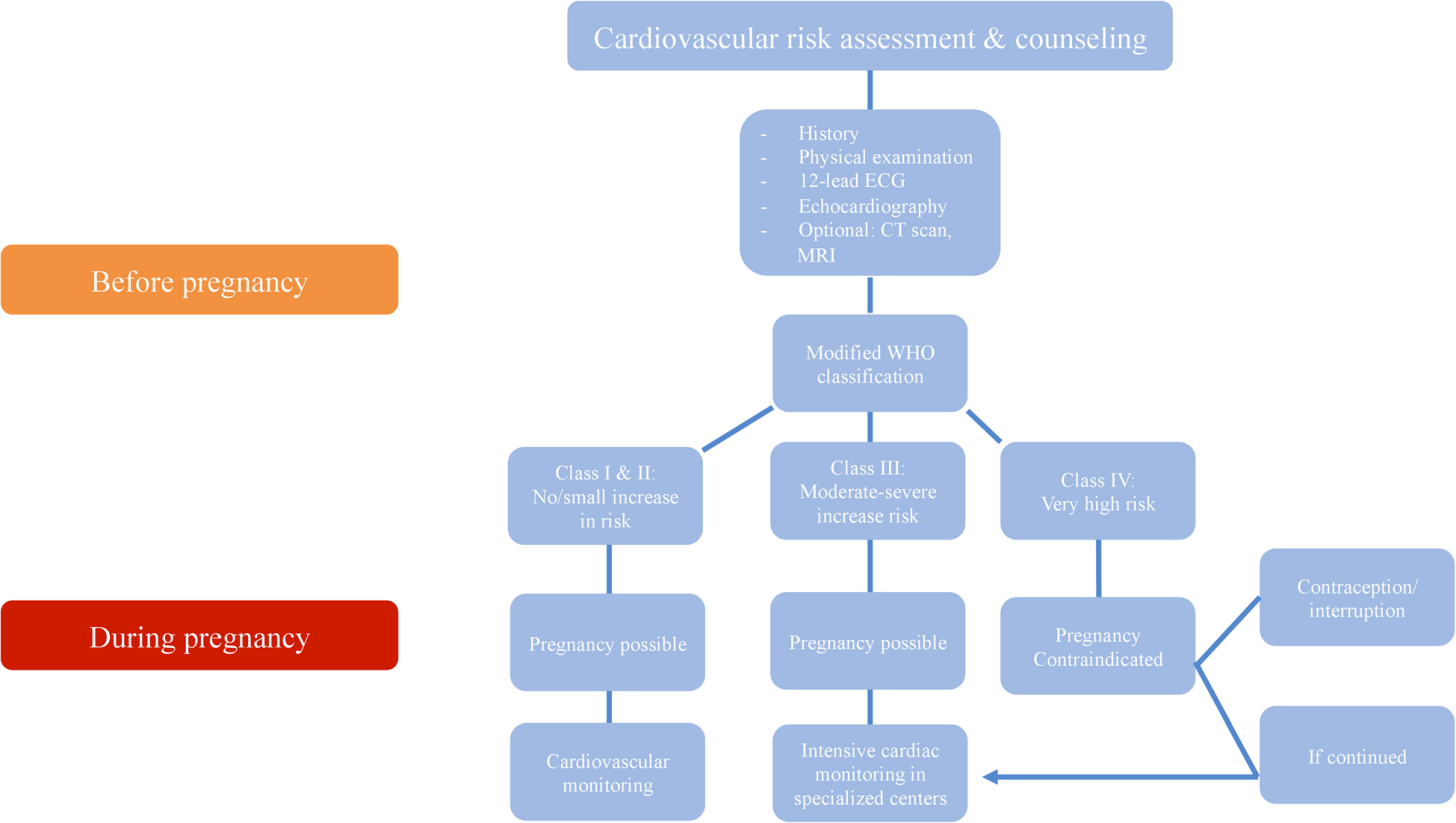
Management of pregnancy and delivery in women with heart disease. ECG = electrocardiogram, CT = computed tomography, MRI = magnetic resonance imaging

Severe asymptomatic aortic stenosis, as present in the patient of this case, is classified as WHO class III, with a 19–27% risk of maternal cardiac events. Expert counseling, monthly follow-ups during pregnancy, and delivery in a specialized center are advised [4]. Figure 6 describes the management of pregnancy and delivery in women with heart disease.

Pregnancy is generally well tolerated in patients with moderate or severe asymptomatic aortic stenosis, whereas symptomatic severe stenosis patients are more likely to require hospitalization. The most common complications are: heart failure, pulmonary edema, and arrhythmias [15, 16]. Despite the severity of our patient’s stenosis, she was asymptomatic before conception and maintained a good functional status throughout the pregnancy. Valvular gradients, as measured by echocardiography, can increase during pregnancy due to the physiological hemodynamic changes that occur.

Patients with a history of valve replacement carry a specific risk of prosthetic thrombosis (higher for mechanical valves) and valve deterioration (in the case of biological valves). Bioprosthetic valves, like the one carried by our patient, are usually advised in women of childbearing age because of the lower requirement for anticoagulation therapy [17, 18].

Although no RCTs have assessed the safety of epidural analgesia in patients with aortic stenosis (vasoplegia is of particular concern in patients highly dependent on pre-load), some case reports exist describing the feasibility of epidural analgesia to facilitate labor and minimize the pain-response in obstetric patients with severe aortic stenosis [19, 20].

The management of induction and labor in a pregnant woman with aortic stenosis depends on the grade of the valvular stenosis (moderate or severe) and the presence of symptoms. In asymptomatic patients with severe aortic stenosis, it is important to consider an individual approach [4]. The woman’s cardiac status, the fetus’s well-being, and the cervix characteristics should all influence the choice and timing of delivery. Although the rate of caesarean section in patients with aortic stenosis is high, at 75%, evidence exists suggesting that vaginal labor should be preferred unless the obstetrician specifically advises against it [15, 21]. Studies comparing methods of delivery in patients with different heart diseases report caesarean section rates to range from 21 to 55% [22, 23]. Moreover, caesarean section is associated with a higher risk of adverse outcomes in the mother [15], earlier delivery, a lower birth weight, major blood loss, higher infection risk, venous thrombosis, and thrombo-embolism [23].

Our case shows that labor induction combined with epidural analgesia in a patient with a severe aortic stenosis is possible. The patient was followed by adequate and continuous hemodynamic and obstetric monitoring; she showed no signs of heart failure, ventricular dysfunction, pulmonary hypertension, or aortic dilation, and demonstrated strong will throughout labor and childbirth.

Because of a favorable initial Bishop score, induction was accomplished by starting with low doses of oxytocin followed by artificial rupture of the membranes at a later stage, showing that these methods of induction can be safe in women with heart disease [4].

**Fig. 7:**
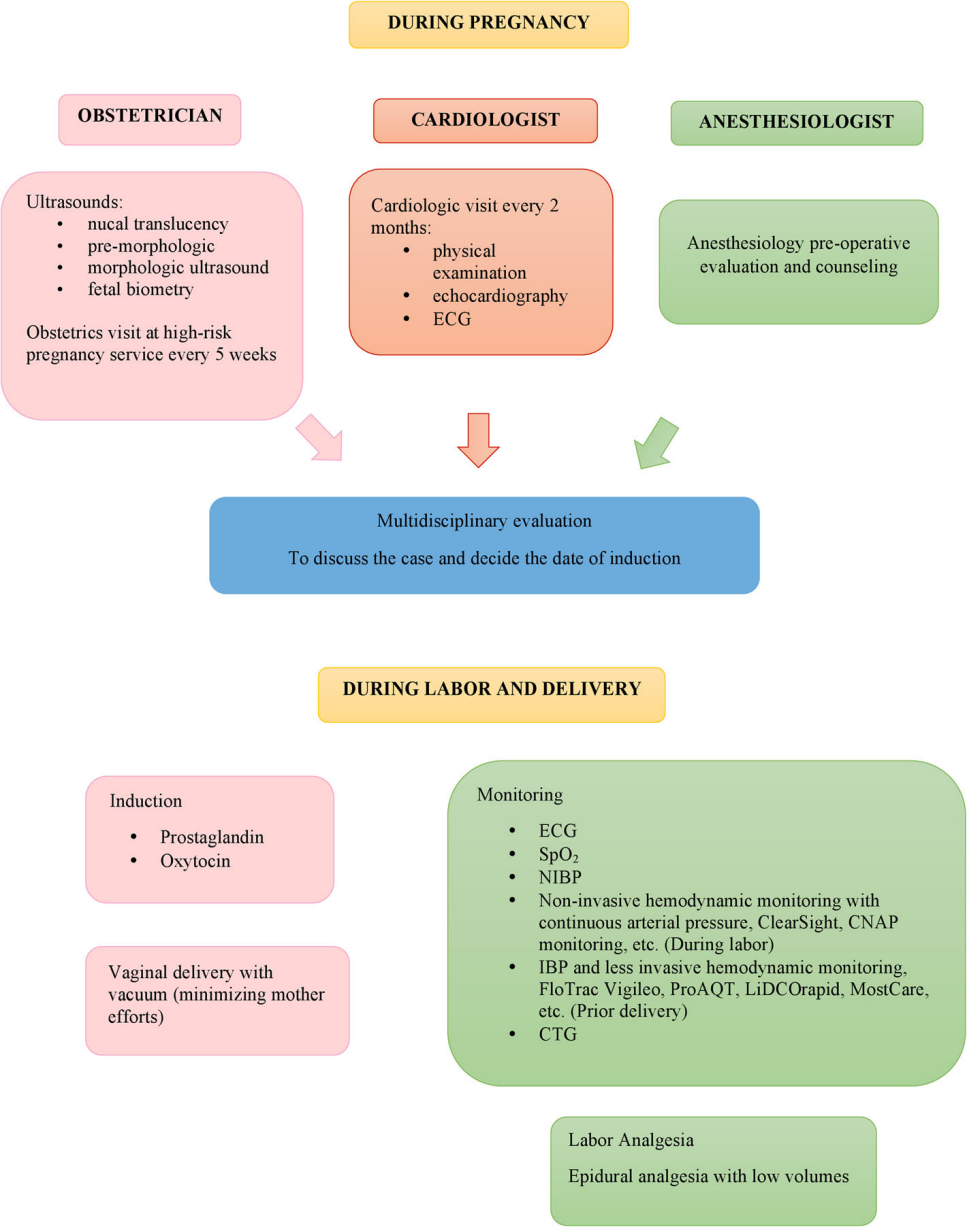
Multidisciplinary clinical management algorithm of pregnancy, labor and delivery in women with heart disease. ECG = electrocardiogram, SpO_2_ = oxygen saturation, NIBP = non-invasive blood pressure, IBP = invasive blood pressure, CTG = cardiotocography

We chose to use the ClearSight™ hemodynamic monitoring system during the first phase of labor to satisfy our requirement for a fast and precise hemodynamic monitoring device that was also non-invasive. For the second stage of labor, we supplemented our system with arterial cannulation and the minimally-invasive FloTrac™ system for beat-by-beat blood pressure and hemodynamic monitoring. This permitted us to allow labor to progress naturally, and uterine contractions alone facilitated the descent of the fetus to the pelvic floor. However, when continuous cardiotocography showed initial alterations, vacuum extraction was performed. Our aim was to minimize the maternal expulsive efforts, which could have worsened her hemodynamic status, and reduced the second stage of labor. We propose a clinical management algorithm, as shown in Fig. 7 [24, 25].

## Conclusion

The case study herein reported demonstrates that epidural analgesia and oxytocin induction are possible for the labor management of parturients with severe aortic stenosis given that continuous non-invasive followed by invasive hemodynamic monitoring can be provided and given the absence of any obstetric or cardiologic contraindications and the strong will of the patient. A protocol for prompt intervention must always be in place to provide for the management of any potential complications. A proactive approach is perhaps the most appropriate for this kind of patient.

## Data Availability

Original data files are available upon request.
